# Two Host Clades, Two Bacterial Arsenals: Evolution through Gene Losses in Facultative Endosymbionts

**DOI:** 10.1093/gbe/evv030

**Published:** 2015-02-20

**Authors:** Pierre-Antoine Rollat-Farnier, Diego Santos-Garcia, Qiong Rao, Marie-France Sagot, Francisco J. Silva, Hélène Henri, Einat Zchori-Fein, Amparo Latorre, Andrés Moya, Valérie Barbe, Shu-Sheng Liu, Xiao-Wei Wang, Fabrice Vavre, Laurence Mouton

**Affiliations:** ^1^Laboratoire de Biométrie et Biologie Evolutive, UMR CNRS 5558, Université de Lyon, Université Lyon1, Villeurbanne, France; ^2^BAMBOO Research Team, INRIA Grenoble, Rhône-Alpes, France; ^3^Institut Cavanilles de Biodiversitat i Biologia Evolutiva, Universitat de València, Spain; ^4^Ministry of Agriculture Key Laboratory of Agricultural Entomology, Institute of Insect Sciences, Zhejiang University, Hangzhou, China; ^5^School of Agriculture and Food Science, Zhejiang Agriculture and Forestry University, Lin’an, Hangzhou, China; ^6^Unidad Mixta de Investigación en Genómica y Salud de la Fundación para el Fomento de la Investigación Sanitaria y Biomédica de la Comunidad Valenciana (FISABIO-Salud Pública) y el Instituto Cavanilles de Biodiversitad y Biología Evolutiva (Universitat de València), Valencia, Spain; ^7^Department of Entomology, NeweYa’ar Research Center, Agricultural Research Organization, Ramat Yishay, Israel; ^8^CEA/DSV/IG/Genoscope, 2 rue Gaston Cremieux, Evry, France

**Keywords:** comparative genomics, *Hamiltonella defensa*, aphids, *Bemisia tabaci*

## Abstract

Bacterial endosymbiosis is an important evolutionary process in insects, which can harbor both obligate and facultative symbionts. The evolution of these symbionts is driven by evolutionary convergence, and they exhibit among the tiniest genomes in prokaryotes. The large host spectrum of facultative symbionts and the high diversity of strategies they use to infect new hosts probably impact the evolution of their genome and explain why they undergo less severe genomic erosion than obligate symbionts. *Candidatus Hamiltonella defensa* is suitable for the investigation of the genomic evolution of facultative symbionts because the bacteria are engaged in specific relationships in two clades of insects. In aphids, *H. defensa* is found in several species with an intermediate prevalence and confers protection against parasitoids. In whiteflies, *H. defensa* is almost fixed in some species of *Bemisia tabaci*, which suggests an important role of and a transition toward obligate symbiosis. In this study, comparisons of the genome of *H. defensa* present in two *B. tabaci* species (Middle East Asia Minor 1 and Mediterranean) and in the aphid *Acyrthosiphon pisum* revealed that they belong to two distinct clades and underwent specific gene losses. In aphids, it contains highly virulent factors that could allow protection and horizontal transfers. In whiteflies, the genome lost these factors and seems to have a limited ability to acquire genes. However it contains genes that could be involved in the production of essential nutrients, which is consistent with a primordial role for this symbiont. In conclusion, although both lineages of *H. defensa* have mutualistic interactions with their hosts, their genomes follow distinct evolutionary trajectories that reflect their phenotype and could have important consequences on their evolvability.

## Introduction

Endosymbiosis is an important evolutionary process in insects, as more than 20% of the insect species depend on endosymbiotic bacteria for their development and survival (Moya et al. 2008; Moran et al. 2008). Insect endosymbioses can be classified based on the host dependency on the bacterium: obligate symbionts are referred to as primary symbionts (P-symbionts), whereas facultative symbionts are called secondary symbionts (S-symbionts). The first are mutualists for the insect, which generally hosts them in specialized cells called bacteriocytes, which in most cases form an organ-like structure known as the bacteriome (Buchner 1965). In most known cases, the classical contribution of such bacteria is nutritional, and involves supplying metabolites that are essential to insect hosts that rely on unbalanced diets like phloem or blood (Buchner 1965; Nogge 1982). These endosymbiotic associations have allowed the hosts to explore new niches, supporting the idea that symbiosis is an important source of evolutionary innovation. As concerns S-symbionts, their prevalence in insect populations is highly variable (Cheng et al. 2000), contrary to the fixed P-symbionts. Their persistence relies on various—not mutually exclusive—strategies including both context-dependent mutualism (Moran, Degnan, et al. 2005; Lamelas et al. 2011) and reproductive manipulation (Stouthamer et al. 1999). S-symbionts can be located in the bacteriocytes but may also be found in different tissues of the host, including the hemolymph (Fukatsu et al. 2000). They are often maternally transmitted, but unlike the P-symbionts whose vertical transmission is strict, S-symbionts are often imperfectly transmitted and can also be horizontally transferred among insects of the same or different species (Chiel et al. 2009). These different dynamics are reflected by the incongruence of phylogenetic trees found between hosts and S-symbionts (Russel et al. 2003; Baumann 2005; Lamelas et al. 2008), as opposed to the nearly perfect cocladogenesis between P-symbionts and their hosts (Baumann 2005). As a consequence of horizontal transfers, insect species harboring P-symbionts can also be infected by S-symbionts, leading to multiple infections within host individuals (Fukatsu et al. 2000; Sloan and Moran 2012b).

The unique lifestyle of symbionts has shaped their genome features. On the one hand, in each generation, only a small part of the symbiotic population is transmitted to the offspring (Moran 1996). These frequent bottlenecks reduce the effective size of the symbiotic population and thus the efficiency of selection with different consequences. Mildly deleterious mutations can accumulate, notably through AT mutational bias (Moran 1996). Similarly, new insertions of mobile elements are less counter-selected, which can later on facilitate intragenomic recombination and large deletions (Silva et al. 2001). On the other hand, different gene categories become dispensable in the new and stable conditions (i.e., the host environment), such as genes related to mobility, sensing of the environment and regulation (Shigenobu et al. 2000). Finally, stability of the association at the intergenerational level favors reduction in the virulence arsenal of symbionts. As a consequence of these different processes and apart from some rare exceptions, endosymbiont genomes have the smallest size and the highest AT bias in eubacteria (Shigenobu et al. 2000; Santos-Garcia et al. 2012; Sloan and Moran 2012a; Lopez-Madrigal et al. 2011). These characteristics are more pronounced in P-symbionts that only retain a gene content close to the minimal set required for life (Gil et al. 2004; Pérez-Brocal et al. 2006). In contrast, S-symbionts that can still infect different tissues and different hosts have retained genes allowing for more versatile behavior. These genes include the ones involved in regulation and environmental perception, and above all virulence factors to overcome the host immune system (Ochman and Moran 2001), some of them being carried by phages and plasmid islands (Wu et al. 2004; Degnan et al. 2009). Although genome differences between P- and S-symbionts are now well documented, it is less clear how specificities in the relationship between the host and S-symbionts can affect the evolution of the bacterial genomes. For example, in a recent genomic study the supply of riboflavin to the host was postulated to be key to the establishment of *Serratia symbiotica* as a co-obligate endosymbiont in aphids from the genus *Cinara* (Manzano-Marín and Latorre 2014).

*Hamiltonella defensa* is a suitable candidate to approach this question. These Gammaproteobacteria can mainly be found in two important clades of insects—aphids and whiteflies (Moran, Russell, et al. 2005; Degnan and Moran 2008b)—and the first data suggest that the role of the bacteria differs in these two hosts. Several aphid species are infected by *H. defensa*, which highlights its ability to horizontally transfer (Russel et al. 2003; Degnan and Moran 2008b). In this clade, *H. defensa* behaves as a protective symbiont against parasitoids, through the synthesis of toxins encoded by the bacteriophage APSE (Moran, Russell, et al. 2005; Oliver et al. 2009). Interestingly, the presence of *H. defensa* is also associated with infection costs (Vorburger and Gouskov 2011), which could explain the intermediate prevalence observed for this symbiont and its correlation with the intensity of parasitoid attacks. Within aphid individuals, *H. defensa* is located not only in the bacteriome, together with their P-symbiont *Buchnera aphidicola* (ensuring its maternal transmission), but also in the insect hemolymph, which may favor contact with the parasitoid (Darby et al. 2001; Moran, Russell, et al. 2005). In whiteflies, contrary to the large host spectrum in the superfamily of aphids, *H. defensa* is restricted to two closely related species of the *Bemisia tabaci* species complex, namely Mediterranean (MED, formerly referred to as the “Q” biotype) and Middle East Asia Minor 1 (MEAM1, formerly referred to as the “B” biotype) species (Gueguen et al. 2010). In addition, *H. defensa* is almost fixed in MEAM1 and in the Q1 subspecies of MED (hereafter named MED-Q1), whereas it is totally absent from the other two MED subspecies Q2 and Q3 (Gueguen et al. 2010; Pan et al. 2012). Also in contrast with the situation in aphids where the symbionts are in separated bacteriocytes (Tsuchida et al. 2005), *H. defensa* coinfects the same bacteriocytes as the P-symbiont *Portiera aleyrodidarum* in *B. tabaci* (Gottlieb et al. 2008; Skaljac et al. 2010). Because *P. aleyrodidarum* is not able to fully compensate for the insect diet and presents some incomplete amino acid biosynthetic pathways, bacteriocyte sharing could favor metabolic exchanges between the two symbionts. In addition, *H. defensa* has been shown to increase growth rate under nutritional stress in the MED species of *B. tabaci* (Su et al. 2014). All these points suggest an important metabolic contribution of *H. defensa* in whiteflies.

Recently, the genomes of *H. defensa* have been sequenced from both aphids and whiteflies. The genome of *H. defensa* from the aphid *Acyrthosiphon pisum* (strain 5AT) exhibits characteristics of an S-symbiont with moderate AT bias and genome reduction (Degnan et al. 2009). Consistent with the phenotype of the bacterium, it contains different virulence factors that may mediate both protection against parasitoids and the capacity to invade the tissues of the insect host. In contrast, the analysis of the *H. defensa* genome from *B. tabaci* MED-Q1 suggests that it could supply its host with different metabolites, including the essential amino acid lysine (Rao, Rollat-Farnier et al. 2015). This is consistent with a primordial role for this symbiont and could explain its quasi-fixation in some whitefly populations. In order to analyze the genomic consequences of the specific lifestyle and relationships *H. defensa* has established with its aphid and whitefly hosts, we compared the genomes of *H. defensa* in *A. pisum* and in *B.tabaci* MED-Q1. The genome of *H. defensa* from the MEAM1 species of *B. tabaci* was also sequenced and compared with the two other genomes. Special attention was given to the history of infection, and to gene content evolution in relation to metabolism, virulence, and environmental sensing. Because of its putative obligate status, it was hypothesized that *H. defensa* in *B. tabaci* would consistently have genomic characteristics and evolution revealing a transition toward primary endosymbiosis.

## Materials and Methods

### Whitefly Strains

A population of *B. tabaci* MEAM1 species reared in laboratory conditions at Newe Ya’ar Research Center (Israel) for many years was used for this study. The population was kept on cotton plants inside insect growth chambers (14–10 h light–dark photoperiod). This strain harbored the P-symbiont *P. aleyrodidarum* as well as the S-symbionts *H. defensa* and *Rickettsia* sp.

### DNA Extraction and Amplification

Approximately 2,000 adult whiteflies were collected. A modified endosymbiont enrichment extraction method was used (Harrison et al. 1989). Whiteflies were homogenized with a Dounce homogenizer in cold Ringer-Krebs buffer (Sigma-Aldrich). The homogenate was passed through a set of decreasing sized porus nylon membranes (1 mm, 80, 60, 20, 10, and 5 μm) (4 °C), centrifuged at 8,000 rpm (revolutions per minute) for 15 min (4 °C) and then endosymbiont pellet was washed with Ringer-Krebs buffer (three times). The pellet containing the endosymbionts was resuspended with 250 μl of Ringer-Krebs buffer and the obtained suspension (445 μl) was subjected to DNase digestion (50 μl of 10× TURBO DNase I Buffer and 5 μl TURBO DNase I) to degrade the host genomic DNA (Harrison et al. 1989), following the manufacturer’s instructions (TURBO DNase, Life Technologies). The final endosymbiont enriched sample was centrifuged at 8,000 rpm for 15 min (4 °C) and washed. The pellet was used for DNA extraction with the JETFLEX Genomic DNA Purification Kit (Genomed) following the manufacturer’s instructions. GenomiPhi V2 (GE Healthcare) was used for genomic amplification following the manufacturer’s recommendations. In order to diminish chimera biases in sequencing data, six independent amplifications were performed and 1 μl of DNA (<10 ng/μl) was used as input for each reaction. Amplified DNA reactions were pooled and used to construct different sequencing libraries.

### Assembly and Annotation

Two libraries were constructed at Genoscope (France): A 454 paired-end library and an Illumina HiSeq 2000 (100 nt). The first assembly was made with mira4 (Chevreux et al. 1999), using the two libraries (both 454 and Illumina reads). BLAST (Basic Local Alignment Search Tool) searches against the NCBI (National Center for Biotechnology Information) databases were performed for each contig to identify which organism it belonged to. Reads coming from other sources (the host *B. tabaci*, the mitochondria, the other symbionts *P. aleyrodidarum*, and *Rickettsia* sp.) were removed. Three assemblies were then performed keeping only the reads from *H. defensa*, with three levels of stringency. CISA (contig integrator for sequence assembly) (Lin and Liao 2013) was used to merge the three obtained assemblies. This step significantly improved the length and gene content of the assembly (data not shown). Mapping was performed with mira4 and SOAPaligner/soap2 (Li et al. 2009) on the final assembly in order to check the quality of the CISA merging. Contig editing and visualization were performed using Gap5 (Bonfield and Whitwham 2010) and Tablet (Milne et al. 2010).

The assembly was deposited on the MaGe platform in order to be annotated (Vallenet et al. 2013). Genes coding for proteins with a length below 100 amino acids were considered as being artifacts if they had no significant matches against MaGe databases, and no identified domains. For the rest, the annotations coming from Swissprot were privileged. Syntenic information was important for inference of the presence of some islands like operons dedicated to the synthesis of fimbriae or siderophores (see Results). The detected CDSs were annotated as pseudogenes when their length was less than 80% of the one of their orthologs in the other genomes, as suggested by Lerat and Ochman (2005), except if all the ancestral domains were preserved. The inactivations and losses specific to our strain of *H. defensa* were checked using SOAPaligner/soap2 software and the Illumina data. The genes broken by contig edges and with no apparent stop mutation were considered as active. The assembly and annotations are available at the European Nucleotide Archive under accession number PRJEB7127.

### Comparative Genomics and Outgroup Genome

We propose a host-based nomenclature to denote the three sequenced genomes of *H. defensa* used in this study (table 1). This nomenclature concatenated the “h” of *Hamiltonella* with the different information about the host taxonomy. The genome we sequenced was thus referred to as hBtab_MEAM1 and the one infecting the MED species of *B. tabaci* as hBtab_MED-Q1 (Rao et al. 2012). We called hApis_5AT the first genome of *Candidatus H. defensa* from *A. pisum* almost entirely sequenced (chromosome with gaps) (Degnan et al. 2009). The two genomes of *H. defensa* of *B.tabaci* will be referred to as the hBtab clade, based on our phylogenetic analysis (see above not only in the Materials and Methods but also in the Results section).

**Table 1:** Nomenclature for the *Hamiltonella defensa* Genomes

Reference	Host Genus	Host Species	Strain (cytotype)	Nomenclature
Degnan et al. (2009)	*Acyrthosiphon* (A)	*pisum* (pis)	5AT	hApis_5AT
Rao et al. (2012)	*Bemisia* (B)	*tabaci* (tab)	MED (Q1)	hBtab_MED-Q1
This study	*Bemisia* (B)	*tabaci* (tab)	MEAM1	hBtab_MEAM1

To study the evolution of the genome in *H. defensa*, several related bacteria were used as outgroups: Two draft genomes of *Regiella insecticola* (Degnan et al. 2010; Hansen et al. 2012), and the genomes of *Yersinia enterocolitica* strain 8081 (Thomson et al. 2006), *Serratia proteamaculans* and *Escherichia coli* strain K12 (Blattner et al. 1997). These genomes represent different levels of divergence with the genus *H. defensa*, all belonging to Gammaproteobacteria: *R. insecticola* is a clade of facultative endosymbionts and the sister species of *H. defensa*, and the three other outgroups are free-living bacteria.

### Gene Families

Blastall was used to blast each protein of a given genome against all the proteins of the seven other genomes. First, large families were built by putting a given protein in the same family of its best hit in each organism, and using a transitive relation (i.e., the respective clustering of A and B on one hand, and A and C on the other hand, means that B and C are in the same family). Thus, two proteins coming from the same genome can be placed in the same family. It allowed the following placements in each family: 1) the fragments of the same protein, 2) the proteins coming from recent duplication events, and 3) the different paralogs coming from both lateral gene transfers and old duplication events. The MUSCLE software (Edgar 2004) was used to perform an alignment of the proteins of each family; PhyML v3.0 (Guindon et al. 2010) was used with default parameter to rapidly build a family tree. These large families were then manually curated, based on several types of information such as alignments and trees. This allowed to obtain more accurate orthologs, and gave a precise idea of the different gene histories which helped in annotating hBtab_MEAM1. Truncated genes, with a length of less than 80% of the one of their homologs, were considered as pseudogenes. Because we focused on the genomic history of the genus *Hamiltonella*, the start codons of the *H. defensa* pseudogenes were checked when the truncation was in the 5′-region in order to eliminate any false positives. For the putative pseudogenes shared by the three *H. defensa* strains, the seqinR package (R) was used to calculate the Ka/Ks ratio between hApis_5AT and the hBtab bacteria in order to determine whether they were still under purifying selection. It was not possible to determine this ratio between the two hBtab bacteria, because of their similarity (close to 100%).

### Reconstruction of the Ancestor of the Genus *Hamiltonella*

The ancestor of the genus *H. defensa* was inferred based on the gene families. A gene was considered as present in the ancestor in two distinct conditions: 1) if the gene is present in both the hBtab clade and the hApis_5AT, and active in at least one of the three genomes of *H. defensa* (a gene present in an inactive state in the three genomes was considered as ancestrally inactivated); and 2) if a gene was intact in either hApis_5AT or the hBtab clade, and present in at least one outgroup genome. This definition means that a transfer between one of the genomes used as outgroup and a genome of *H. defensa* would be erroneously considered as ancestral. To avoid such problems, the mobile elements (phages, plasmid islands, toxin/antitoxin systems, transposases) were removed when studying the ancestor, except for the APSE phage and the genes belonging to the nonintegrated plasmid of hApis_5AT.

### Phylogeny of the Genus *Hamiltonella*

To go further, we built the phylogeny of the genus *Hamiltonella* to determine the relationship between the *H. defensa* of whiteflies and aphids. Eight genes (*accD*, *dnaA*, *gyrB*, *hrpA*, *murE*, *ptsI*, *recJ*, and *rpoS*) have already been sequenced in 19 *H. defensa* of aphids and in 1 *H. defensa* of the MEAM1 species of *B. tabaci* (Degnan and Moran 2008b). Among these eight genes, six are 100% identical to their respective ortholog in the genome of hBtab_MEAM1 sequenced in this study (*accD*, *dnaA*, *gyrB*, *hrpA*, *murE*, and *ptsI*), and five of these six genes are 100% identical to their orthologs in hBtab_MED-Q1. Moreover, these six genes are clearly distinct from the orthologs of the *H. defensa* of aphids. The two remaining genes (*recJ* and *rpoS*) exhibit a different pattern: in the MEAM1 strain of *H. defensa* sequenced in Degnan and Moran (2008b), they are totally identical to their orthologs in some *H. defensa* of aphids, and distinct from their orthologs in the genome of the two assembled genomes of *H. defensa* of *B. tabaci* (hBtab_MEAM1 and hBtab_MED-Q1). These two genes are unlikely to have been submitted to frequent transfers (see, e.g., their individual HOGENOM gene trees HOG000018414 and HOG000270273; Penel et al. 2009), and the similarity between the *H. defensa* of *B. tabaci* and aphid species for these two loci could reflect sequencing issues rather than horizontal transfers. In all cases, they probably do not reflect the phylogenetic history of the species, and were thus removed from the analysis.

Thus, six genes were used to study the phylogenetic relationships between the genomes of *H. defensa* in aphids and *B.tabaci* (*accD*, *dnaA*, *gyrB*, *hrpA*, *murE*, and *ptsI*). We used both the sequences from Degnan and Moran (2008b), to which we added the corresponding sequences in the three assembled genomes of *H. defensa*, and two outgroups (*R. insecticola* strain 5.15 and *Y. enterocolitica* strain 8081). Genes were individually considered and then concatenated.

For each set of genes, alignments were initially generated using the MUSCLE software (Edgar 2004) and then implemented in the CLC Main Workbench v6.7.1 (CLC Bio). Phylogenetic analyses were performed using maximum-likelihood inference with PhyML v3.0 (Guindon et al. 2010). The appropriate model of evolution was evaluated with jModeltest v0.1.1 (Posada 2008) for each set of sequences and the best likelihood score was evaluated with the Akaike information criterion (AIC) for the concatenated data sets and AIC corrected for small sample size for data of gene separately. The models selected were TPM + I + G for *accD*, TVM + I + G for *gyrB*, TVM + G for *hrpA* and *dnaA*, TPM3 + G for *murE*, TIM3 + G for *ptsI*, and GTR + G for the concatenated data sets. Robustness of the nodes was assessed with 100 bootstrap replicates. Finally, the trees were edited with Figtree v1.4.0 (A. Rambaut, http://tree.bio.ed.ac.uk/software/figtree). *Yersinia enterocolitica* strain 8081 was used to root the tree.

## Results

### Genome Features of hBtab_MEAM1

The genome of hBtab_MEAM1 was assembled into 183 contigs. The total length of the assembled genome is 1.72 Mb (table 2), but this is probably an underestimation of the real size of the genome as it includes some multicopy sequences that were partially collapsed during the process. However, all the unique sequences in the genome are probably represented in the assembly. The coverage is indeed on average 100-fold, which should guarantee a representation of a large part of the genome. Furthermore, the assembly contains the sequences of the 205 genes known to be universally present as single-copy genes in almost all Gammaproteobacteria (Lerat et al. 2003). The persistence of multiple contigs thus most probably reflects the abundance of repetitive sequences, which represented a problem for the assembly. In conclusion, this assembly allows for a confident interpretation of the gene functions present in *H. defensa.*

**Table 2:** Properties of Different Draft/Complete Published *Gammaproteobacteria* Genomes

Features	*Buchnera aphidicola* APS	hBtab_MEAM1	hBtab_MED-Q1	hApis_5AT	*Regiella insecticola* LSR1	*Escherichia coli* K12	*Yersinia pestis* CO92
Chromosome size	640,681	1,726,317	1,840,000	2,110,331	2,035,106	4,639,221	4,653,728
Extrachromosomal element	2	0	0	1	1	0	3
Total G + C (%)	26.2	40.1	40.3	40.1	42.4	50.8	47.6
Predicted CDS	571	1,400	1,806	2,100	1,761	4,284	4,012
Pseudogenes	13	232	NA	188	214	150	149
Average CDS size	984	965	NA	812	856	950	998
Coding density (%)	86.7	78.8	NA	80.8	71.4	87.9	83.8
rRNA operons	2	2^a^	NA	3	4	7	6
tRNAs	32	40	38	42	36	86	70

Genome status	Complete	Draft	Draft	Complete	Draft	Complete	Complete
Reference	Shigenobu et al. (2000)	This study	Rao et al. (2012)	Degnan et al. (2009)	Degnan et al. (2010)	Blattner et al. (1997)	Parkhill et al. (2001)

Note.—NA, missing data.

^a^In hBtab_MEAM1, the real number of rRNA operons is not known: One operon contains the three rRNA genes (5S, 23S, and 16S), and there is also one copy of the 16S rRNA and one copy of the 5S rRNA which are each near the end of a different contig. It is not possible to determine whether these two copies are on the same operon, and whether there is a second 23S copy or not.

Overall, the general characteristics of hBtab_MEAM1 are very similar to those of both hBtab_MED-Q1 and hApis_5AT in terms of size, gene, and GC content (table 2). All three genomes are typical of S-symbionts, showing moderately reduced size and AT bias, and a high number of repetitive sequences compared with the total absence in long-term P-symbionts. The most important difference between hApis_5AT and the two *H. defensa* strains from *B. tabaci* is the probable absence of the nonintegrated plasmid pHD5AT in the hBtab clade. In hBtab_MEAM1, 1,400 coding sequences (CDS) and 232 pseudogenes were identified, which represents a slightly lower gene number compared with the other *H. defensa* genomes (table 2). The differences between the two close genomes of hBtab_MED-Q1 and hBtab_MEAM1 could be due to differences in the respective assembly, that is, in the parameters that have been applied for CDS detection and in pseudogene annotation, but they also reflect some real differences in gene content. The most telling example concerns an entire (inactivated) 15-kb-long *tri* operon, present in both hBtab_MED-Q1 and hApis_5AT, but absent from the genome of hBtab_MEAM1.

### Phylogenetic Reconstruction and Infection History

**Fig. 1.—:**
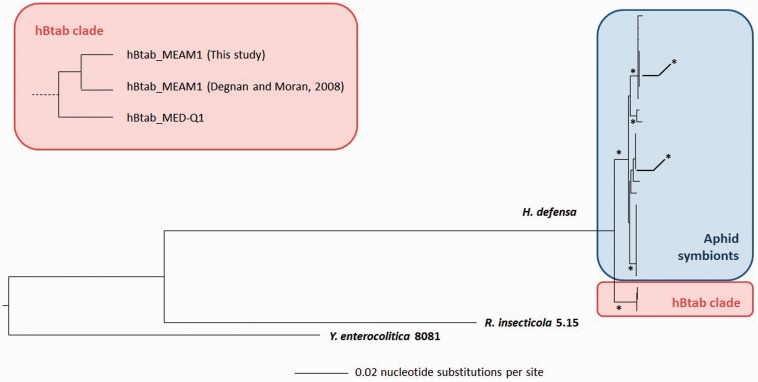
Simplified phylogeny of the genus *Hamiltonella.* Bootstrap values greater than 80 are represented by an asterisk. The monophyly of the hBtab clade is supported by a concatenation of six gene sequences and a bootstrap value of 100. A zoom on the hBtab clade illustrates the separation between the symbionts of the MEAM1 and MED-Q1 species.

In order to clarify the phylogenetic relationships between the strains associated with aphids and whiteflies, we concatenated six orthologous loci from 23 *H. defensa* strains, among which 20 were infecting aphids and three infecting *B. tabaci.* The phylogenetic analysis showed that the three bacteria of *B. tabaci* form a monophyletic clade (fig. 1), hereafter referred to as the hBtab clade. The phylogenetic proximity between the bacteria of *B. tabaci* is consistent with the 99.9% of similarity at the level of the 16S rDNA gene between the MEAM1 and MED-Q1 strains, which each have 98% similarity with the sequence of hApis_5AT. Moreover, the comparative genomic analysis clearly revealed different events of gene inactivations and acquisitions specific to each clade (see below).

Due to their genomic divergence, the presence of *H. defensa* in aphids and whiteflies can only be explained by horizontal transfers (Darby et al. 2001). Within the *B. tabaci* species complex, the two *H. defensa* from MEAM1 are monophyletic (fig. 1), but only one difference distinguishes them from MED-Q1 for the six used loci. The divergence between the 16S rDNA sequences of *H. defensa* MED-Q1 and MEAM1 is of the same order of magnitude as the 99.8% of divergence between the sequences of *P. aleyrodidarum* MED-Q1 and MEAM1 (Santos-Garcia et al. 2012). Because the presence of *P. aleyrodidarum* in the *B. tabaci* MED/MEAM1 ancestor is certain, the hypothesis of an ancestral presence of *H. defensa* cannot be excluded. Moreover, the genomic differences between *H. defensa* MED and MEAM1 are sufficient to exclude a very recent horizontal transmission, and suggest the existence of two distinct strains. In conclusion, even though the *H. defensa* of MED-Q1 and MEAM1 are separated, it is still difficult to determine whether *H. defensa* was present in their ancestor or independently acquired by the two insects species/biotypes.

### Gene Content Evolution in *H. defensa*

After the removal of pseudogenes, fragments due to the draft assembly, duplications, and small ORFs without any significant match in the TrEMBL and Swiss-Prot databases, the orthology relationship between the three *H. defensa* strains revealed an important set of common genes (supplementary fig. S1, Supplementary Material online). The substantial difference in global gene number and content compared with previous studies (Degnan et al. 2009; Rao et al. 2012) stems from the strict cleaning step, which eliminated several hundreds of genes, especially those specific to the hBtab clade or to hApis_5AT. For example, among the genes specific to hApis_5AT, more than 300 were eliminated because they were composed of less than 100 amino acids and had no significant match in the database. More than 100 were pseudogenes, and more than 200 were eliminated because they were associated with duplication events.

To further analyze the gene contents of the *H. defensa* genomes, we reconstructed the putative ancestor of this genus based on the gene content of all *H. defensa* strains and one of their Gammaproteobacteria relatives. The reconstruction of the ancestor highlighted that the acquisition of new genes represents a small proportion of the functional differentiation between the bacteria. Indeed, the genes specific either to the hBtab bacteria or to hApis_5AT are mostly mobile elements and are located on inactive phage and plasmid islands. They were thus removed from the analysis in order to focus on the functional aspects. It then appeared that the new acquisitions of genes are restricted to operons which are dedicated to the synthesis of sugars and which are specific of each lineage, Type 3 effectors and RTX toxins (details below). The great majority of the observed differences thus comes from different losses/inactivations from the 1,265 genes composing the ancestral gene content rather than from massive gene acquisitions (supplementary fig. S1, Supplementary Material online).

Gene losses have been more numerous in the hBtab clade (Pearson’s test, *P* value < 0.05), where they have affected many functions known to be important for the ecology of hApis_5AT, such as virulence factors or two-component systems (TCSs) (Degnan et al. 2009). However, despite its protective role, hApis_5AT lost many virulence factors retained in the hBtab clade. Some of the most important losses in each clade are detailed below.

### Functional Losses in the hBtab Clade

#### Extrachromosomal Elements

The hApis_5AT strain is associated with a conjugative IncFII plasmid called pHD5AT, present both in nonintegrated and integrated forms (Degnan et al. 2009). Although the nonintegrated form remains intact in hApis_5AT, the integrated form is inactive due to the absence of some genes (*traQ*, *traR*) and the inactivations of others (*traY*, *traT*, *traC*, *pilL*, *pilN*, plus the two *repA* genes).

Several genes of the pHD5AT plasmid are absent in the hBtab clade (*traLMNOPQRT*) and only a fragment of the *repA* gene is found in these genomes, making the replication of any nonintegrated plasmid impossible. As a consequence, the plasmid genes of this clade are probably inactive islands integrated in the bacterial chromosome. Consistently, a large plasmid region (corresponding in hApis_5AT to the region between HDEF_p0016 and HDEF_p0053) is surrounded in hBtab_MEAM1 by two genes which are found in the bacterial chromosome of hApis_5AT but absent from its plasmid. Similarly, the above-mentioned fragment of *repA* (corresponding to HDEF_p0001) is found in the neighborhood of chromosomal genes in the two bacteria of the hBtab clade. The most probable scenario is thus an ancestral chromosomal integration and inactivation of the plasmid in the genus *Hamiltonella*, followed by the loss of the nonintegrated plasmid in the hBtab clade.

The APSE phage is another well-characterized partner of the *H. defensa* genus (Moran, Degnan, et al. 2005). Different inactivations occurred in the hBtab phage and concerned the major head protein P24, the *kilA*-domain protein G, the regulator protein I, and the P38_2 integrase, which allows both phage integration and excision. Thus, as for the conjugative plasmid, the APSE phage is probably inactive in hBtab. In spite of this inactivation, the APSE phage in hBtab encodes a CdtB toxin that retains the 12 active residues described in Degnan and Moran (2008a), and which is involved in the protection against parasitoids in aphids (Moran, Degnan, et al. 2005; Degnan and Moran 2008a).

In addition, many small phage and plasmid islands are found in the hBtab genomes, like in hApis_5AT (Degnan et al. 2009) where they have been supposed to be inactive. As in hApis_5AT, these islands contain a large proportion of pseudogenes, and are probably inactive and progressively degraded.

#### Type 3 Secretion Systems

**Fig. 2.—:**
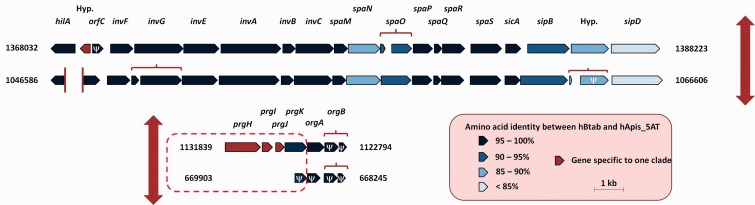
SPI-1 (T3SS) gene clusters in hApis_5AT (top) and hBtab_MEAM1 (bottom). The vertical red arrow represents an ancestral split of the operon. The vertical red bars indicate contig boundaries. Genes are referred by their name, except hypothetical genes (Hyp.). Pseudogenes are indicated by the letter **ψ**. Some genes are fragmented but are not considered as pseudogenes, according to our parameters of detection. The different fragments of a gene are linked by a red curly bracket. The *orgB* ancestral inactivation could have already reduced the efficiency of this system in the last common ancestor. In hBtab, *prgH*, *I*, *J*, and *K* are absent or inactive, leading very certainly to a nonfunctional system (red dashes).

Two Type 3 secretion systems (T3SS) have been described in hApis_5AT (Degnan et al. 2009). They were called SPI-1 and -2 (for “*Salmonella* Pathogenicity Island”) because their organization was similar to the secretion systems of *Salmonella.* These two T3SS are largely degraded in hBtab, especially the SPI-1-like system (fig. 2). Some genes directly involved in the apparatus formation are missing or have become pseudogenized, making this system probably inactive.

The SPI-2 T3SS also contains pseudogenes in the hBtab clade. Indeed, the TCS SsrAB, which is involved in the activation of T3SS and is directly linked to virulence (Okan et al. 2006; Okan et al. 2010), is inactive due to a *ssrA* pseudogene. In addition, the *ssaK* gene, essential for the SPI-2 apparatus (Niemann et al. 2011), has been independently truncated in the two hBtab bacteria. Even though there is not as many gene losses as for SPI-1, the fact that two mutations independently occurred in a structural gene of the SPI-2 system suggests a relaxation of the selective pressure. This system may therefore also be non- or subfunctional in the hBtab clade.

Consistent with its active T3SS, hApis_5AT retains several Type 3 effectors that are absent or pseudogenized in the hBtab genomes (Degnan et al. 2009; table 3). All these effectors confer virulence and invasiveness to pathogenic bacteria and could be related either to protection against parasitoids or to evasion of the host immune system. This is the case of the *yopH* and *yopT* genes, which are important for resistance against phagocytosis (Andersson et al. 1996; Grosdent et al. 2002). Interestingly, *H. defensa* has recently been shown to persist in phagocytes in aphids (Schmitz et al. 2012). In contrast, the hBtab genomes possess a single well-characterized Type 3 effector that is absent in hApis_5AT: The SPI-1 associated effector YopJ/P. *Yersinia* uses YopJ to induce cell death, inhibiting the host immune response by blocking the MAPK (Mitogen-Activated Protein Kinase) and NFκB pathways (Orth 2002). It has also been presented as a candidate for protection against parasitoids in *R. insecticola* (Hansen et al. 2012). However, the fact that the SPI-1 system is inactive in hBtab raises the question of its role in this clade, as is also the case for the three effectors that are shared between all *H. defensa.*

**Table 3:** Type 3 Effectors in the Genomes of the *Hamiltonella defensa* Genus

Gene	hBtab_MEAM1	hBtab_MED-Q1	hApis_5AT
*ssaE*	Intact	Intact	Intact
*ssaB*	Intact	Intact	Intact
*exoY_2*	Intact	Intact	Intact
*yopJ*	Intact	Intact	Absent
*aexT_1*	Absent	Absent	Intact
*aexT_2*	Absent	Absent	Intact
*exoY_1*	Absent	Absent	Intact
*nleD*	Absent	Absent	Intact
*yopT_1*	Absent	Absent	Intact
*yopT_2*	Absent	Absent	Intact
*yopH*	Pseudogene	Pseudogene	Intact

In conclusion, the virulence arsenal within the hBtab clade is greatly reduced compared with hApis_5AT, with both inactivation of the T3SS and loss of numerous effectors.

#### Environmental Perception

SsrAB is not the only TCS truncated in the hBtab clade (supplementary fig. S2, Supplementary Material online). Indeed, two other systems involved in environmental perception have been inactivated, one in each hBtab strain (supplementary fig. S2, Supplementary Material online). The UvrY/BarA TCS is an important regulator of the metabolism, also controlling quorum-sensing, motility, and the production of toxins (Lapouge et al. 2008). It is well conserved among Gammaproteobacteria in which it coordinates pathogenicity and social behavior. In hBtab_MEAM1, BarA is fragmented into three, whereas in hBtab_MED-Q1, the gene seems intact, but its reconstruction is not complete as it is cut by the end of a contig. The other system is the ExpRI-like quorum-sensing system, which was ancestrally present in *H. defensa*, but is inactive in hBtab_MED-Q1 (*expI* is absent and *expR* inactive). Finally, the case of ArcAB is more complicated. ArcAB is a global regulator modulating more than 100 genes involved in central metabolism and respiratory pathways (Martínez-Antonio and Collado-Vives 2003). It also allows for resistance against nitrogen species and reactive oxygen species in aerobic conditions (Lu et al. 2002). The ancestor *R. insecticola*/*H. defensa* probably already underwent a mutation in ArcB: Compared with its orthologs in Gammaproteobacteria, the sequences in these symbionts lack the PAS domain, which is an oxygen/light sensor (Taylor and Zhulin 1999). Nevertheless, the Ka/Ks ratio of 0.45 between the hBtab bacteria and hApis_5AT reveals that it stayed under purifying selection after this mutation. However, in hBtab, a second mutation introduced a premature stop codon, making the gene probably inactive.

Altogether, these results suggest a lower response of the hBtab clade to environmental variations compared with the symbiont of aphids.

#### Metabolism of Cofactors

**Fig. 3.—:**
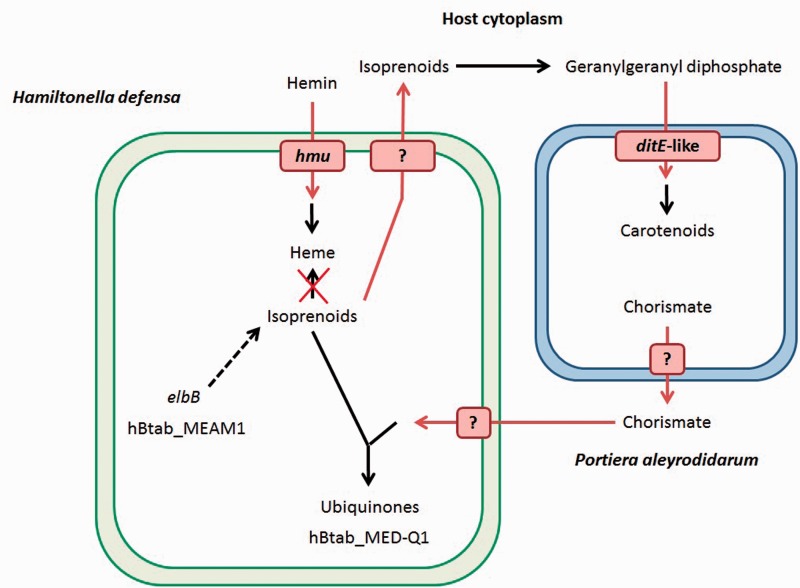
Isoprenoid fate in the hBtab clade. The rectangle on the left represents the hBtab bacteria, the rectangle on the right *P. aleyrodidarum.* Dark arrows indicate biosynthetic pathways. Red arrows indicate metabolite exchanges. The red cross indicates that the two bacteria of the hBtab clade are unable to use isoprenoid to produce heme, and have to uptake the latter. In hBab_MEAM1, the dashed arrow illustrates the enhancement of the synthesis of isoprenoids by the *elbB* gene. hBtab_MED-Q1 might depend on the chorismate furnished by *P. aleyrodidarum* to synthesize ubiquinones. Isoprenoids are produced by the two bacteria of the hBtab clade, and the surplus could be furnished to the other partner for the production of carotenoids. Concerning transporters, the hmu system and the ditE-like gene have been, respectively, identified by Degnan et al. (2009) and Rao, Rollat-Farnier et al. (2015). No transporter for isoprenoids was identified. Concerning chorismate, which is a dicarboxylate, sodium:dicarboxylate symporters could allow its transport.

hApis_5AT can synthesize different cofactors such as biotin, pyridoxal-5-phosphate, coenzyme A, or heme, through the import of host metabolites such as glucose or pantothenate (Degnan et al. 2009). The hBtab clade has a metabolism close to the one of hApis_5AT, but different inactivations prevent the production of some cofactors. An example is protoheme, an intermediary compound in the biosynthesis of the heme, which cannot be produced by the two bacteria of the hBtab clade due to many ancestral gene inactivations (*hemBCDEFGL*). However, the hemin transport system (*hmuRTSUV* operon) still allows the bacterium to import host hemin, which can be spontaneously converted into heme. In addition, hBtab_MED-Q1 is unable to produce chorismate due to an inactivation in *aroA.* These gene inactivations could allow the host to control (and restrict) the utilization of isoprenoids by hBtab. Indeed, heme contains an isoprenoid chain, and chorismate is used with isoprenoids to produce ubiquinones. This is interesting because isoprenoids are the precursors of carotenoids, important metabolites that the P-symbiont *P. aleyrodidarum* supplies to its host (Santos-Garcia et al. 2012; Sloan and Moran 2012a). In the same way, hBtab_MEAM1 possesses a gene which is lost in hBtab_MED-Q1 and hApis_5AT (*elbB*) which could favor isoprenoid synthesis and thus enhance carotenoid production (Hemmi et al. 1998). Thus, in the two *H. defensa* strains of *B. tabaci*, in addition to the common inability to synthesize heme, two strategies could have emerged to preserve/enhance isoprenoid production (fig. 3).

### Functional Losses in hApis_5AT

#### Type 2 Secretion System

Contrary to the hBtab clade, hApis_5AT has an inactive Type 2 secretion system (T2SS) with inactivated *gspE* and *gspF* genes combined with a split of the T2SS in two regions of the genome. The T2SS system is also associated with virulence (Korotkov et al. 2012), and controlled in some species by quorum-sensing (Sandkvist 2001). In the closely related genus of *Yersinia*, T2SS has been much less studied than T3SS but could be associated with cell invasion and/or with survival outside of the mammalian hosts (von Tils et al. 2012).

#### Alpha-Fimbriae Synthesis

The fimbriae of the alpha-fimbriae family are involved in the adherence of enterotoxigenic *E. coli* and the colonization of the human intestine (reviewed in Nuccio and Bäumler 2007). Several cryptic fimbriae are found in *E. coli*, and are differentially expressed depending on the environmental conditions. hBtab could have two such systems that are inactive in hApis_5AT. Indeed, one pseudogene of hApis_5AT is localized in a cluster of five genes identified as encoding proteins involved in the formation of alpha-fimbriae in *Yersinia pestis* CO92 (Parkhill et al. 2001). This cluster would encode a fimbrial major subunit, a fimbrial minor subunit, two fimbrial chaperones, and a fimbrial usher protein. The minimum required set of proteins is composed of the major subunit, the chaperone, and the usher protein (Nuccio and Bäumler 2007), the latter being inactive in hApis_5AT. A second cluster contains four genes that could be involved in another alpha-fimbriae formation. It would encode a protein with an unknown function, an usher protein, a major subunit, and a chaperone. The two first genes, and therefore also the usher protein, are inactive in hApi_5AT, leading to a nonfunctional system.

#### Siderophore Synthesis

**Fig. 4.—:**
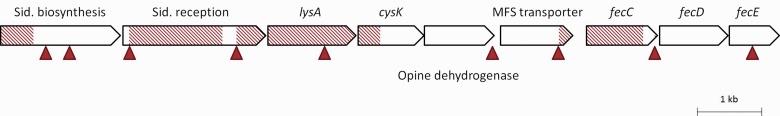
Siderophore biosynthesis gene cluster in the hBtab clade. The mutations that occurred in the different orthologs of hApis_5AT are indicated by red markers: Stop mutations are indicated by triangles, whereas gene deletions are represented by hatches. Several inactivations occurred in this bacterium: Only *fecD* was found intact, and only this gene and the opine dehydrogenase were not considered as pseudogenes with our parameters.

hApis_5AT underwent several inactivations of genes involved in iron uptake that are intact in hBtab. These inactivations include two genes involved in the synthesis of siderophore, a gene involved in the reception of siderophore, and two genes—*fecC* and *fecE—*annotated as iron transporters (Degnan et al. 2009). All these genes are in the same island (fig. 4), which has only been characterized in the pathogen *Proteus mirabilis* (Himpsl et al. 2010), where it synthesizes a newly described kind of siderophore, the proteobactin (*ptb*). The Fec-like iron transporter would serve to the siderophore uptake, once this last has chelated iron (Himpsl et al. 2010). This cluster is upregulated in iron-limiting conditions, but mutations in this cluster do not decrease the bacterium’s ability to infect hosts (Himpsl et al. 2010).

#### Metabolism of Amino Acids

As seen in figure 4, the *pbt* island contains two genes, *cysK* and *lysA*, involved in the metabolism of cysteine and lysine, respectively, which are inactive in hApis_5AT. A second *lysA* gene is present in hBtab, but is also inactive in hApis_5AT. *Buchnera aphidicola*, the P-symbiont associated with hApis_5AT, is able to produce both cysteine and lysine. Thus, the inactivation of such genes in hApis_5AT could be explained by ancestral metabolic redundancies. On the other hand, both hBtab bacteria retained the ability to synthesize these amino acids, which has been lost in *P. aleyrodidarum* from *B. tabaci.*

### Common Conservation and Loss of Virulence Systems

Two virulence systems, the Tad gene cluster and T1SS, have undergone similar evolution in both clades. First, the Tad gene cluster that encodes a type IVb pilus notably involved in adhesion and host colonization (Kachlany et al. 2001) has been independently inactivated by pseudogenization in both clades: *rcpA* in hBtab (plus *tadC* in hBtab_MED-Q1) and *tadV* in hApis_5AT. These independent inactivations would be one of the rare convergences observed between the hBtab clade and hApis_5AT.

Second, T1SS is intact both in hApis_5AT (Degnan et al. 2009) and in the hBtab clade. This system is involved in the secretion of the RTX toxins (Holland et al. 2005), of which several copies are present in the *H. defensa* genomes (table 4). Although quite a number of different genes encode RTX toxins in all three genomes, many of them are pseudogenized. Considering the ancestral pool of RTX toxins probably present in the ancestor of the three genomes, some differential inactivations occurred (table 4). The three bacteria only retained two common toxins, a putative serralysin and rtxA_09, which is fragmented in hApis_5AT but whose largest fragment is longer than the threshold we used to characterize pseudogenes.

**Table 4:** RTX Toxins in the Three Genomes of *Hamiltonella defensa*

Gene	hApis_5AT	hBtab_ MEAM1	hBtab_ MED-Q1
HDEF_0400	Intact	Intact	Intact
rtxA_08–rtxA_09	Intact (Fg)	Intact	Intact
rtxA_03	Intact	Contig	Contig
rtxA_27	Intact (Fg)	Intact	Contig
rtxA_14	Intact (Fg)	ψ	ψ
rtxA_07	Intact (Fg)	ψ	ψ
rtxA_16	Intact	Absent	Absent
rtxA_22–HDEF_1932	ψ	Intact	Intact
rtxA_28–rtxA_29	ψ	Contig	Contig
rtxA_21	ψ	Contig	Contig
HBTMEAM1_v1_820005	Absent	Intact	ψ
rtxA_33	Absent	Contig	ψ
HBTMEAM1_v1_1390001	Absent	Contig	ψ
HDEF_2014–rtxA_25	ψ	Intact	ψ
rtxA_30–rtxA_32	ψ	ψ	Intact
rtxA_15	ψ	ψ	ψ
rtxA_17	ψ	ψ	ψ
HDEF_0620–rtxA_05	ψ	Absent	Absent
HDEF_0633–rtxA_06	ψ	Absent	Absent
HDEF_1149	ψ	Absent	Absent
HDEF_1222	ψ	Absent	Absent
HDEF_1264–HDEF_1267	ψ	Absent	Absent
HDEF_1389	ψ	Absent	Absent
rtxA_01–rtxA_02	ψ	Absent	Absent
rtxA_10–rtxA_12	ψ	Absent	Absent
rtxA_13	ψ	Absent	Absent
rtxA_18–rtxA_20	ψ	Absent	Absent
rtxA_26	ψ	Absent	Absent

Note.—Fg, fragmented gene, but whose longest fragment is still considered active according to our parameter (see Materials and Methods); ψ, pseudogene; Contig, gene with no visible deleterious mutation but not entire due to assembly.

### Rapid Evolution and Divergence of Cell Wall Components

Cell wall components are highly different in hBtab and hApis_5AT due to both specific gene losses, and the presence of two operons that probably originate from specific acquisitions.

**Fig. 5.—:**
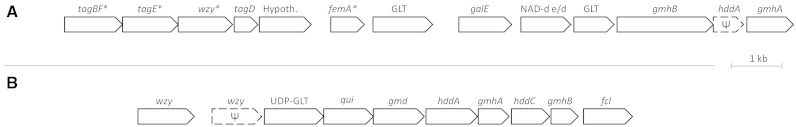
Gene clusters involved in the biosynthesis of sugars and cell components specific to hApis_5AT (*A*) and the hBtab clade (*B*). The names of the genes are accompanied by an asterisk when the attribution is only based on the presence of conserved domains (and some syntenic clues for the *tag* genes). Pseudogenes are indicated by dashed lines and the **ψ** letter. GLT, glycosyltransferase; Hypoth., hypothetical; NAD-d e/d, NAD-dependent epimerase/dehydratase. hApis_5AT contains intact *gmhA* and *gmhB* genes, distantly related to the hBtab copies, but no functional *hddC* and *hddA* genes, so that dd-Hep production is not possible in this bacterium.

This is especially visible as concerns sugar biosynthesis. On one hand, hApis_5AT harbors an entirely original gene cluster dedicated to the synthesis of cell wall components (HDEF_0185 to HDEF_0197, fig. 5*A*). It notably contains the *tagD* gene and two genes with *tagE* and *tagB*/*tagF* signatures. The *tag* gene cluster allows the synthesis of teichoic acids (TA) in Gram-positive bacteria, a heterogeneous class of phosphate-rich polymers covalently linked to the cell wall peptidoglycan. TAs may serve as a reservoir of phosphates to prevent starvation (Grant 1979) and as phage receptors (Chatterjee 1969). They also allow bacterial adhesiveness and antigenic power (Wicken and Knox 1975). Because its organization and gene content differ from those of Gram-positive bacteria (reviewed in Swoboda et al. 2010), we can make the assumption of an original gene pathway in hApis_5AT. Apart from the above-mentioned gene cluster, hApis_5AT possesses an active copy of *gne*, which allows the production of UDP-*N*-acetyl-α-d-galactosamine (GalNAc). GalNAc has been found in *Enterobacteriaceae* as a component of the O-antigen of the lipopolysaccharide, or LPS (Bengoechea et al. 2002; Liu et al. 2008), and in the teichoic acid of *Bacillus subtilis* (Young 1966).

On the other hand, hBtab also possesses original genes for sugar biosynthesis. In particular, one hBtab operon may have been acquired through horizontal transfer, as no trace of ortholog genes has been found in hApis_5AT. It contains two genes involved in the biosynthesis of GDP-l-fucose (l-Fuc) from GDP-d-mannose (*gmd* and *fcl*) (fig. 5*B*)*.* The hBtab bacterium also encodes the *qui* gene, presumed to be involved in the biosynthesis of GDP-l-quinovose (l-Qui) from GDP-l-Fuc (De Castro et al. 2013). Only two bacteria are known to possess l-Qui, both in their O-antigen fraction: *Yersinia pseudotuberculosis* serotype O:12 (De Castro et al. 2013) and *Providencia alcalifaciens* O:44 (Kocharova et al. 2005). All the genes (*gmhA*, *gmhB*, *hddA*,** and *hddC*) allowing the synthesis of d-glycero-d-manno-heptose (dd-Hep) are also present in this operon (fig. 5*B*).

Another potentially important difference between the two clades concerns the O-antigen, which is the more distal component of LPS, and is composed of different sugars linked together. It is a common factor of the Gram-negative bacteria that is important for host–pathogen interactions (Skurnik et al. 2000). Four genes are necessary to perform its assembly/export: *wzx*, *wzy*, *wzz,* and *rfaL* (Skurnik et al. 2000). In hApis_5AT, we identified putative candidates for these four genes: 1) HDEF_0181, which possesses a weak similarity to the *wzx* flippase; 2) HDEF_0195, homolog of *wzy*; 3) *fepE* (HDEF_0537), which has a role analogous to *wzz* in *Salmonella typhimurium* (Murray et al. 2003); and 4) HDEF_2072, which contains a *rfaL*-like O-antigen ligase domain. These results suggest that hApis_5AT is able to produce O-antigen, which could be a TA-like O-antigen, as already identified in several Gram-negative bacteria (Jann et al. 1980; Perepelov et al. 2006).

**Fig. 6.—:**
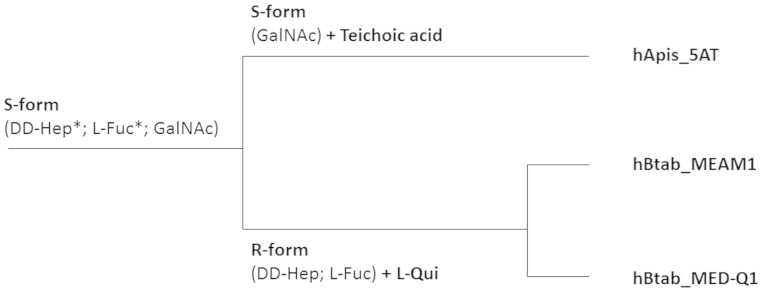
O-antigen and putative LPS-associated sugars in *H. defensa.* The ancestor was a putative S-form bacterium, whereas the hBtab clade could group together with R-form bacteria due to the inactivation of a *rfaL*-like gene. The sugars that generally bind to the O-antigen fraction are indicated in the corresponding branch and are detailed in the section “Rapid Evolution and Divergence of Cell Wall Components.” Asterisks represent sugar pathways that could be ancestral (i.e., with close and best homologs in *Yersinia* species or in *R. insecticola*), but that could also have been horizontally acquired by the hBtab clade.

In contrast, in hBtab, an inactivation occurred in the *rfaL*-like gene, which generally leads to rough-type bacteria (R-form), that is, to bacteria without O-antigen. Moreover, in hBtab_MED-Q1, no homolog of *wzy* was found. The presence of both O-antigen assembly genes and different pathways of sugar biosynthesis (fig. 6) suggests that the ancestor of *H. defensa* was an S-form bacterium, that is, harboring an O-antigen, and that this O-antigen has been specifically lost in the hBtab clade. This result is surprising as hBtab is able to produce l-Qui, l-Fuc, and dd-Hep, which are sugars of the O-antigen of Enterobacteriacea, and especially of the *Yersinia* species (reviewed in Bruneteau and Minka 2003). However, all these sugars are also found in other cell components, as could be the case in hBtab.

## Discussion

Recent studies have suggested that *H. defensa* could have a nutritional role in *B. tabaci.* Indeed, both genomic and experimental results sustain a metabolic role of this symbiont, which could occur through the complementation of the P-symbiont *P. aleyrodidarum* not only for both provision of vitamins and cofactors but also for production of essential amino acids. Through comparison with the genome sequence of the defensive *H. defensa* infecting aphids, our goal was to study the impact of the ecology of the host–symbiont interaction (extended phenotype, within-host localization, etc.) on the evolution of the gene content of the symbiont. The sequence of the *H. defensa* genome from *B. tabaci* MEAM1 allowed comparison with the genomes of *H. defensa* in aphids and another species of *B. tabaci*, and to infer the gene content of their ancestor.

### An *Yersinia*-Like Arsenal for a Pathogenic Ancestor

The genome of hApis_5AT already revealed an important number of virulence factors (Degnan et al. 2009), and our analysis allowed extension of the characterization of the virulence islands present in the ancestor of *H. defensa.* The ancestral genome probably harbored at least three intact secretion systems (T1SS, T2SS, and T3SS), several pili/fimbriae (type IV pili, alpha-fimbriae), different toxins and effectors, two extra-chromosomal partners (the APSE phage and a plasmid), different systems of environmental perception, a siderophore, and a putative O-antigen linked to different sugars. Many of these systems have been well described in the *Yersinia* species and are significant virulence factors for this genus. This is in agreement with the theory that various virulence factors associated with pathogenic bacteria are part of the genomic repertoire of symbionts as they are necessary to overcome the host immune system (Hentschel et al. 2000; Degnan et al. 2009).

### Divergence through Gene Losses

An initial observation is that genome evolution has mainly been driven by gene loss in both lineages (table 5). Indeed, very few genes seem to have been acquired through horizontal gene transfer (table 5). This trend is even stronger in the hBtab clade, where the possibility of DNA uptake could be reduced, due to the loss and/or inactivation of the extrachromosomal elements, which participate in gene transfers. Importantly, the losses are highly divergent between the hBtab clade and hApis_5AT, except for some RTX toxins and the Tad cluster. This suggests that the ancestor of *H. defensa* was already a symbiont having experienced genome reduction, as suggested (Degnan et al. 2010). Indeed, many studies have highlighted the fact that the ecological switch from a free-living bacterium to a symbiotic lifestyle generally leads to convergences in the gene losses, due to a similar relaxation of the selective pressures (McCutcheon and Moran 2010; Lamelas et al. 2011). These divergent losses thus reflect the specialization of *H. defensa* to two different clades of insects—aphids and whiteflies.

**Table 5:** Ecological and Genomic Characteristics of Two Clades of *Hamiltonella defensa*

Symbiont Characteristics		hBtab Clade	hApis_5AT
Nature of the relationship	Infected host	*Bemisia tabaci* (whitefly)	*Acyrthosiphon pisum* (aphid)
	Phenotype	Synthesis of essential elements?	Protection
	Prevalence	High (quasifixation)	Medium
	Tissue specificity	Important (primary bacteriocytes)	Weak (whole insect body)
Specific losses	Symbiotic partners	APSE phage and pHD5AT plasmid	
	Metabolism	Heme	Lysine and cysteine
	Environment perception	TCSs	
	Secretion	Type III secretion systems	Type II secretion system
	Other virulent power	O-antigen	Siderophore—Fimbriae
Independent acquisitions	Cell wall components	Sugar biosynthesis (fucose, quinovose, etc.)	Teichoic-acid-like O-antigen?
	Type 3 effectors	yopJ	aexT_1, aexT_2, exoY_1, nleD, yopT_1, yopT_2

Specialization of endosymbionts to different hosts has already been reported, especially concerning the well-documented *B. aphidicola* species. Charles et al. (2011) highlighted that the set of transporters in the genomes of *B. aphidicola* depends on the host plant use: The more specialized the host, the more devoid of transporters the symbiont genome. Moreover, a comparative genomic study of four *Buchnera* strains, each associated with a different host, revealed 80% of divergent gene losses (Latorre et al. 2005), in agreement with the retention of metabolic capabilities related to the host diet/life cycle. Concerning *H. defensa*, between the symbionts of whiteflies and aphids, we did not find a massive divergence of the metabolism, except that hApis_5AT lost genes involved in the lysine and cysteine pathways. This could be the consequence of the metabolic ability of the associated P-symbiont, *B. aphidicola*, to produce these amino acids, in contrast to the situation in *B. tabaci* where *P. aleyrodidarum* has lost part of these pathways. Although only a few genes are involved in this metabolic difference between the two genomes, they might be extremely important for the global evolution of the system. Indeed, in *B. tabaci*, participation to the synthesis of amino acids might be one of the major contributions of the symbiont to the extended phenotype of the holobiont (i.e., the host and its symbiotic community).

Finally, in some aphids of the genus *Uroleucon*, the endosymbiont *H. defensa* could be obligate (Degnan and Moran 2008b). It would be of great interest to study their genomes and to determine whether their genomic evolution follows the same trajectory as the symbionts of the hBtab clade, due to their putative common obligate status.

### Two Bacteria, Two Arsenals

It appears in our analysis that the majority of the differences between the gene repertoires of hApis_5AT and the hBtab clades involved virulence factors. Virulence factors in *H. defensa* might be related to two different processes. First, they can be involved in the interaction with the host and notably the escape of the immune system by the symbiont. Second, they can target natural enemies of the host, especially in *H. defensa* that is a protective symbiont in aphids (Moran, Russell, et al. 2005). Given that *H. defensa* could be a nutritional symbiont in whiteflies and that it colonizes only a single tissue devoted to symbiont hosting, attenuation of the virulence arsenal was expected in this lineage.

A first indication of this attenuation is the loss of the two ancestral T3SS in hBtab, in contrast to their retention in hApis_5AT. T3SS is probably used by hApis_5AT not only to colonize the different tissues of the insect but also to invade new hosts during the phases of horizontal transfers. Consistently, it has been shown that aphid-infecting *H. defensa* are able to resist phagocytosis in the hemolymph (Schmitz et al. 2012). T3SS is the best candidate to confer this phenotype. More generally, T3SS is probably among the most pathogenic weapons of *Enterobacteriaceae*, and especially of the close *Yersinia* species (Cornelis 2002). In addition, many known S-symbionts use T3SS to establish symbiosis with their host (Dale and Moran 2006), even though some, like *Wolbachia*, only harbor a T4SS (Moya et al. 2008). It has thus been proposed that the loss of T3SS could follow the evolution toward a mutualistic and obligate relationship (Dale et al. 2005; Sloan and Moran 2012b), even though some P-symbionts still possess a T3SS (Dale et al. 2002). Another difference between the two *Hamiltonella* lineages concerns the composition of the cell wall. Not only the O-antigen would have been lost in the hBtab clade, but the lineages would have independently acquired different pathways for cell wall sugar synthesis. These variations may account for important differences in the immune dialog between the host and the symbiont. In particular, absence of the O-antigen could limit the ability of *H. defensa* to live outside the bacteriocytes in *Be. tabaci*, as it may participate in the evasion of phagocytosis (Saldías et al. 2009).

Although the above examples highlight an attenuation of the virulence in hBtab, other systems have been conserved in this lineage, whereas lost in hApis5_AT, such as the T2SS system, the production of siderophores, and of fimbriae. However, in several symbiotic associations, “virulence” factors that are classically used for pathogenicity induction play an important role in the establishment and maintenance of symbiosis (Hentschel et al. 2000). As such, T2SS is mandatory for gut colonization by *Aeromonas veronii*, a mutualist symbiont of the leech (Maltz and Graf 2011). The fact that few virulence factors are shared between the two lineages (except for T1SS) makes it probable that communication between the host and the symbiont is highly different in the two systems.

Contrary to what is found in aphids, *H. defensa* in whiteflies does not possess an active APSE phage, suggesting that it does not induce protection against parasitoids. However, the genes encoding for the CdtB toxin are still present in the hBtab clade and exhibit a perfect conservation of the important amino acid residues (Degnan and Moran 2008a). If sequestration within the bacteriome does not preclude protection, as suggested in other symbiotic systems (Nakabachi et al. 2013), secretion of this toxin could allow the targeting of parasitoids. Another possibility, explaining the maintenance of this toxin, is that in addition to its protective role, it could distend the host cytoplasm to facilitate the symbiont’s establishment (Moran, Degnan, et al. 2005).

Finally, even though virulence attenuation seems important in hBtab, it still conserves many virulence factors whose function remains elusive. Two hypotheses could account for their maintenance. First, phylogenetic inertia can be sufficient to explain the presence of many virulence factors. Second, they might be involved in the molecular dialog between the host and the symbiont.

### Versatility and Self-Sufficiency

Confinement to a single specialized cell-type, that is, the bacteriocytes of whiteflies, was expected to favor the loss of genes involved in environmental perception in the hBtab clade. The loss of genes encoding TCSs and quorum-sensing in hBtab indeed reveals symbionts with weakened abilities to cope with environmental variations compared with hApis_5AT. Strict intracellular localization can also explain the relaxation of selective pressure on the pathways involved in the synthesis of compounds available within the cell. Loss of the ability to synthesize heme, an important metabolite for bacterial respiration, could be such an example. Most of the accessible heme is indeed found intracellularly, and is thus directly available for the symbiont. In contrast, hApis_5AT, which invades different tissues and the hemolymph, has retained the ability to synthesize it.

All these features indicate that, compared with the ancestor and the actual hApis_5AT, hBatb is much more dependent on the stable environment provided by the bacteriocyte and on metabolites procured by the host. Such increased dependence could allow tighter control of the symbiotic population by the host, in terms of both localization and density, as has been proposed for *B. aphidicola* in aphids (Thomas et al. 2009; Shigenobu and Wilson 2011).

### Mutualism and the Evolution of Evolvability

Reductive evolution of P-symbionts has often been associated with the idea that these organisms have reduced evolvability (Silva et al. 2003). A comparison of the genomes of the two lineages, both mutualistic but where the advantage provided by the symbiont is either nutritional or protective, provides an interesting opportunity to study how the extended phenotype constrains the evolution of evolvability.

In aphids, the protective phenotype provided by *H. defensa* requires the ability of the symbiont to both circulate within the host body and kill the parasitoid, which maintains a high selective pressure on the virulence systems. In addition, because the symbiont is engaged in an arms race with the parasitoid, the maintenance of mechanisms allowing a rapid adaptation could also be under strong selection. The conservation of extrachromosomal elements favoring horizontal gene transfers and the rapid turnover of genes encoding toxins and virulence effectors in the protective *H. defensa* of aphids support this view. In agreement with the arms race theory, the different APSE phages have been involved in the exchange of toxins between close strains of *H. defensa* in aphids (Moran, Degnan, et al. 2005; Degnan and Moran 2008a). All these features may have different evolutionary consequences. First, maintenance and turnover of virulence factors required for the protective effect may avoid a complete reduction of the infection cost for the host, which could also be associated with the impossibility to reach high frequency for the symbiont. Second, the versatility of the symbiont may allow it to regularly acquire new hosts through horizontal transmission.

In contrast, the transition toward nutritional symbiosis in whiteflies might have considerably modified the selective pressures acting on the symbiont. Indeed, nutritional provision must have been associated with a dramatic increase in the prevalence of the symbiont within populations, thus increasing the selection on the holobiont for a reduction in the infection cost. Such reduction may occur through the loss of virulence factors and/or through the restriction of the localization of the symbiont within the host. Importantly, this could also be associated with the loss of the protective phenotype if it was ancestral. Both the reduction of the arms race with the parasitoid and the confinement within bacteriocytes could have then led to the degradation of extrachromosomal elements*.* Confinement can also favor the evolution of an increased dependency on host metabolites. All these features would rapidly lead to organisms with highly reduced evolvability and ability to switch hosts.

## Conclusions

Both phylogenetic and phylogenomic analyses revealed a clear separation of the *H. defensa* of *B. tabaci* and aphids*.* This is congruent with the genome content of the bacteria, with specific gene acquisitions and losses. Interestingly, the evolution is mainly driven by gene inactivations and losses, whereas horizontal gene transfers seem restricted to some toxins, effectors, and sugars linked to the cell wall. The different gene inactivations that occurred in the symbionts of *A. pisum*, on one hand, and *B. tabaci*, on the other, can reflect the specialization of the bacteria in two distinct host clades as well as the different phenotypes and life conditions. Indeed, hApis_5AT would need some highly virulent factors, such as T3SS, and different environmental perceptors, in order to invade host tissues, transfer to the next generation, and protect the host against parasitoids. These systems would be obsolete in the bacteriome-associated symbionts of the hBtab clade. Nevertheless, the latter retained different virulence factors lost in hApis_5AT. Because a protective phenotype has not yet been demonstrated in the hBtab clade, these factors could have an important role for the communication of *H. defensa* with the host and/or the primary endosymbiont, and/or to settle in the host cells. Globally, although both lineages have mutualistic interactions with their hosts, the evolution of their genomes shows different trends that could have important consequences on the evolvability of these organisms.

## Supplementary Material

Supplementary figures S1 and S2 are available at *Genome Biology and Evolution* online (http://www.gbe.oxfordjournals.org/).

Supplementary Data
