# Supplementary material for: Two Host Clades, Two Bacterial Arsenals: Evolution through Gene Losses in Facultative Endosymbionts
Source: Genome Biol Evol. 2015 Feb 20;7(3):839–55. doi: 10.1093/gbe/evv030 (PMC5322557; doi:10.1093/gbe/evv030)
Supplement: Supplementary Data [file supp_7_3_839__index.html]

Two host clades, two bacterial arsenals: evolution through gene losses in facultative endosymbionts — Two Host Clades, Two Bacterial Arsenals: Evolution through Gene Losses in Facultative Endosymbionts — Supplementary Data 

# Two Host Clades, Two Bacterial Arsenals: Evolution through Gene Losses in Facultative Endosymbionts

## Supplementary Data

files

**Files in this Data Supplement:**

- Supplementary Data - jpg file
- Supplementary Data - jpg file
- Supplementary Data - docx file
