# Supplementary figures and images for: Two Host Clades, Two Bacterial Arsenals: Evolution through Gene Losses in Facultative Endosymbionts
Source: Genome Biol Evol. 2015 Feb 20;7(3):839–55. doi: 10.1093/gbe/evv030 (PMC5322557; doi:10.1093/gbe/evv030)

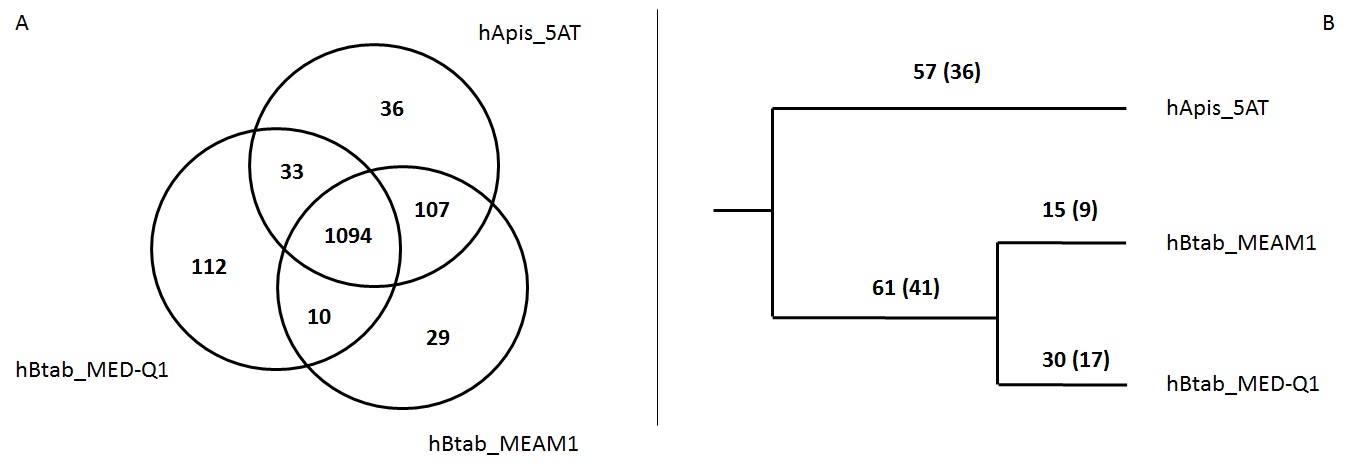

Supplement: Supplementary Data [file supp_evv030_Figure_S1_v3.jpg]

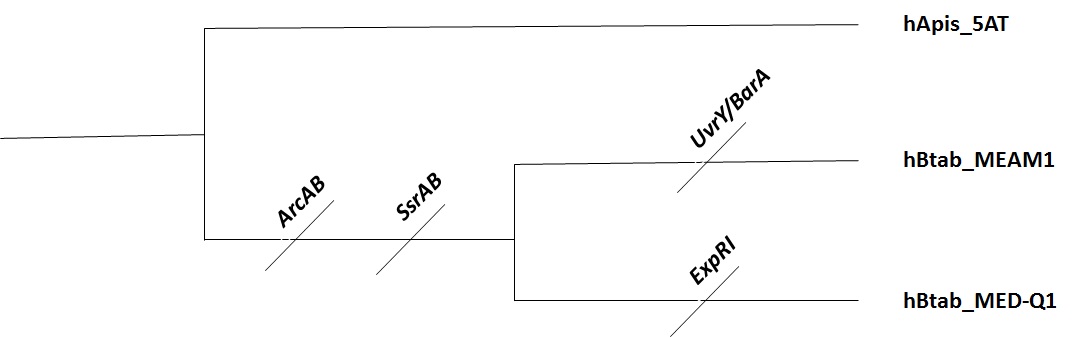

Supplement: Supplementary Data [file supp_evv030_Figure_S2_v3.jpg]
