# Supplementary material for: Two Host Clades, Two Bacterial Arsenals: Evolution through Gene Losses in Facultative Endosymbionts
Source: Genome Biol Evol. 2015 Feb 20;7(3):839–55. doi: 10.1093/gbe/evv030 (PMC5322557; doi:10.1093/gbe/evv030)
Supplement: Supplementary Data [file supp_evv030_New_Microsoft_Office_Word_Document.docx]

**Figure S1.** (A) Euler diagram of the orthologs shared by the three bacterial strains after manual curation of the gene families and a cleaning step. (B) Gene losses (including pseudogenes) in the *H. defensa* clade are represented on the corresponding branches. Genes from phage and plasmid islands have been removed.

**Figure S2.** Putative inactivation of the two-component and quorum-sensing systems along the evolution of *H. defensa*. The bars represent inactive systems.
